# Identification of Candidate Genes and Pathways in Dexmedetomidine-Induced Cardioprotection in the Rat Heart by Bioinformatics Analysis

**DOI:** 10.3390/ijms20071614

**Published:** 2019-04-01

**Authors:** Yusuke Yoshikawa, Naoyuki Hirata, Hirofumi Terada, Yasuaki Sawashita, Michiaki Yamakage

**Affiliations:** Department of Anesthesiology, Sapporo Medical University School of Medicine, South 1, west 16, Chuo-ku, Sapporo, Hokkaido 060-8543, Japan; naohirata@mac.com (N.H.); hirofumi_tera_hirofumi@yahoo.co.jp (H.T.); ya.su.a.ki.ki.a.su.ya@gmail.com (Y.S.); michiaki_yamakage@icloud.com (M.Y.)

**Keywords:** dexmedetomidine, reperfusion, microarray, preconditioning

## Abstract

Dexmedetomidine (DEX), a highly selective alpha2 adrenergic receptor agonist, directly protects hearts against ischemia/reperfusion (I/R) injury. However, the detailed mechanism has not been fully elucidated. We studied differentially expressed mRNAs and miRNAs after DEX administration in rat hearts by comprehensive analysis. Additionally, bioinformatics analysis was applied to explore candidate genes and pathways that might play important roles in DEX-induced cardioprotection. The results of microarray analysis showed that 165 mRNAs and 6 miRNAs were differentially expressed after DEX administration. Through bioinformatics analysis using differentially expressed mRNAs, gene ontology (GO) terms including MAP kinase tyrosine/serine/threonine phosphatase activity and pathways including the p53 pathway were significantly enriched in the down-regulated mRNAs. *Dusp1* and *Atm* were associated with the GO term of MAP kinase tyrosine/serine/threonine phosphatase activity and the p53 pathway, respectively. On the other hand, no significant pathway was found in the target mRNAs of deregulated miRNAs. The results indicated some possible key genes and pathways that seem to be of significance in DEX-induced cardioprotection, although miRNAs seem to be unlikely to contribute to cardioprotection induced by DEX.

## 1. Introduction

Cardiac ischemia/reperfusion (I/R) injury can occur in various clinical situations including the perioperative period of cardiac or non-cardiac surgery. Therefore, much attention has been paid to the selection of anesthetics during the perioperative period. The cardioprotective effects of volatile anesthetics have been investigated in both animal experiments [1,2,3,4] and clinical trials [5,6,7,8], and the American College of Cardiology/American Heart Association guideline [9] recommends volatile anesthetics for the maintenance of general anesthesia in coronary artery bypass graft surgery.

Dexmedetomidine (DEX), which is a highly selective alpha2 adrenergic receptor agonist, is frequently used as a sedative agent in the operating room and intensive care unit in daily clinical practice. We and others have shown that DEX directly protects hearts against I/R injury without autonomic nervous system modulation in both normal healthy hearts and diseased hearts [10,11,12,13,14,15,16]. The cardioprotective effect of DEX has been shown in multiple clinical studies as well as in animal experiments [17,18,19,20]. Hence, the use of DEX is considered to be a novel and promising strategy for cardioprotection during the perioperative period. Regarding the mechanism of DEX-induced cardioprotection, we and others have shown that activation of extracellular signal-regulated kinase (ERK) 1/2 and endothelial nitric oxide synthase (eNOS) is involved in cardioprotection by DEX [10,14,15]. It is generally considered that ERK1/2 is the most traditional mitogen-activated protein kinase (MAPK) and that eNOS is regulated by MAPK activation [21,22,23]. However, the detailed mechanism has not been fully elucidated.

MicroRNAs (miRNAs) are 21–23 nucleotide endogenous non-coding RNAs that negatively regulate target genes at the post-transcriptional level by cleavage or translational repression of target mRNAs [24]. Several miRNAs have been suggested to be associated with biological and pathological processes including cardiac I/R injury [25,26,27], and with the carioprotective effects of preconditioning interventions [28,29]. Regarding volatile anesthetic preconditioning by isoflurane, for instance, it was reported that isoflurane protected the heart against I/R injury via up-regulation of miR-21 [30,31]. Although some previous studies demonstrated that DEX exerted organ protective effects via several miRNAs in the brain [32,33], liver [34], and lung [35], the role of cardiac miRNA in DEX-induced cardioprotection against I/R injury has not been studied.

The aim of the present study was to identify the differentially expressed mRNAs and miRNAs after DEX administration in rat hearts by comprehensive analysis. Based on the results of bioinformatics analysis, we propose some candidate genes and pathways that might play crucial roles in DEX-induced cardioprotection against I/R injury.

## 2. Results

### 2.1. DEX Administered before Ischemia Exerted a Cardioprotective Effect against I/R Injury In Vivo

**Figure 1:**
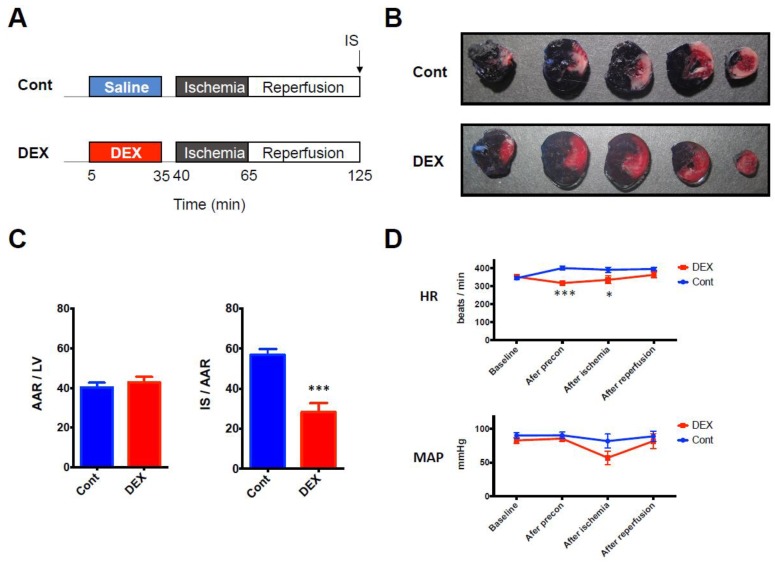
(**A**) I/R experimental protocol. (**B**) Representative LV cross sections. (**C**) Corresponding analysis of AAR expressed as a percentage of LV and IS expressed as a percentage of AAR. (**D**) Hemodynamic parameters during the experiment. *N* = 7 in each group. * *p* < 0.05, *** *p* < 0.001. AAR: area at risk, Cont: control group, DEX: dexmedetomidine group, HR: heart rate, I/R: ischemia reperfusion, IS: infarct size, LV: left ventricle, MAP: mean arterial pressure.

Firstly, we confirmed the cardioprotective effect of DEX against I/R injury by using an in vivo rat model of I/R injury (Figure 1A). DEX administered before ischemia for 30 min significantly decreased infarct size (IS) compared to that in the control group (28% ± 5% in the DEX group vs. 57% ± 3% in the control group, *p* = 0.0002) (Figure 1B,C). Hemodynamic parameters were also measured at 4 time points (baseline, end of DEX preconditioning, end of ischemia, and after reperfusion). Although DEX temporarily decreased heart rate (HR) at the end of preconditioning (318 beats/min ± 12 beats/min in the DEX group vs. 400 beat/min ± 10 beats/min in the control group, *p* = 0.0005) and at the end of ischemia (335 beats/min ± 21 beats/min in the DEX group vs. 391 beats/min ± 14 beats/min in the control group, *p* = 0.028), there was no significant difference after reperfusion. DEX did not alter mean arterial blood pressure (MAP) at any time point (Figure 1D).

### 2.2. Identification of Differentially Expressed mRNAs and miRNAs after DEX Preconditioning

**Figure 2:**
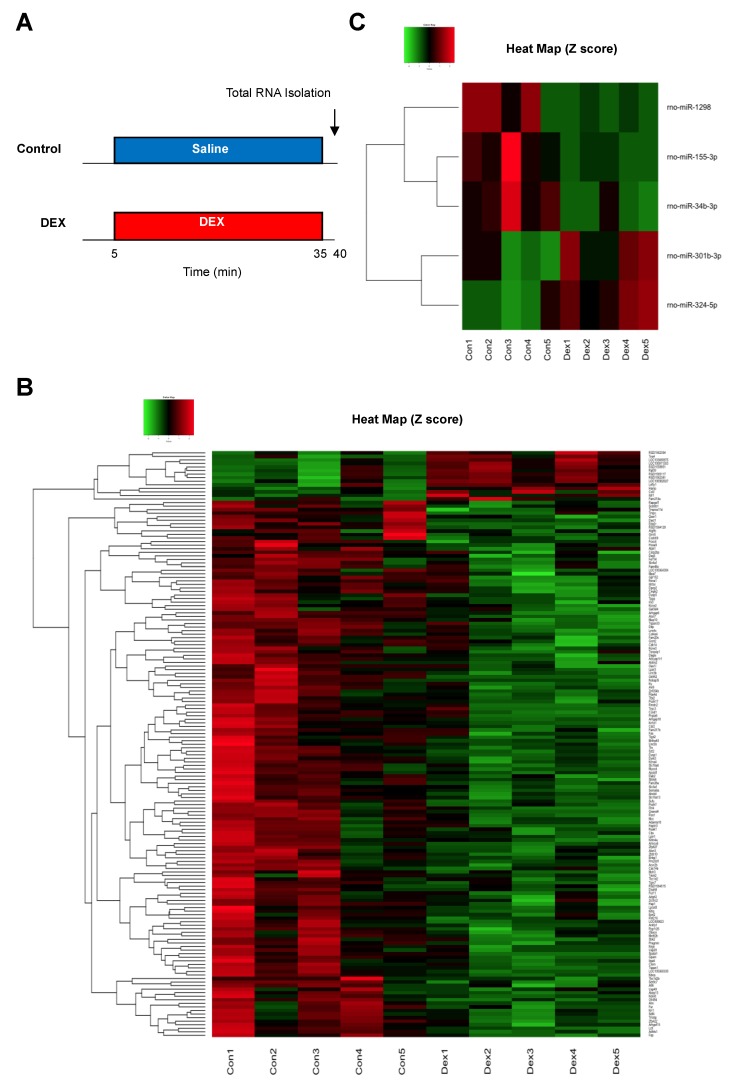
(**A**) Experimental protocol for analysis of differentially expressed mRNAs and miRNAs. (**B**) Heat map of differentially expressed mRNAs after administration of DEX. (**C**) Heat map of differentially expressed miRNAs after administration of DEX. The red and green colors represent >1.5-fold change (red: up-regulated genes, green: down-regulated genes). *N* = 5 in each group. DEX: dexmedetomidine.

**Figure 3:**
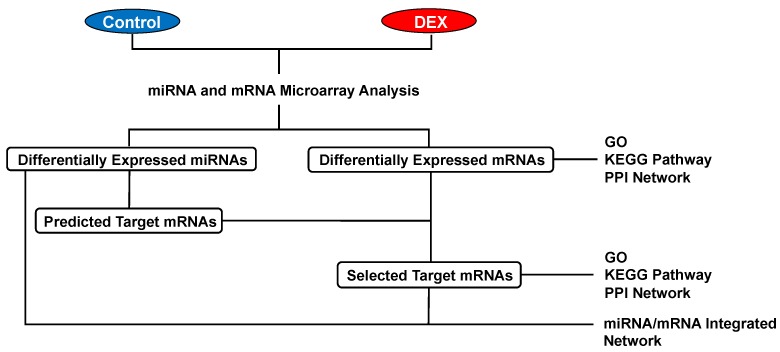
Multi-step approach for analysis of the differentially expressed mRNAs and miRNAs. DEX: dexmedetomidine, GO: gene ontology, KEGG: Kyoto Encyclopedia of Genes and Genomes, PPI: protein-protein interaction.

Differentially expressed mRNAs and miRNAs in rat hearts were identified after DEX administration for 30 min (Figure 2A). In our mRNA microarray analysis, a total of 165 mRNAs (14 up-regulated and 151 down-regulated mRNAs) were differentially expressed after DEX preconditioning compared to controls (Tables S1 and S2). In the miRNA microarray analysis, only 6 miRNAs (3 up-regulated and 3 down-regulated miRNAs) were identified as differentially expressed miRNAs after DEX preconditioning (Table 1). The expression levels of these mRNAs and miRNAs are presented by a hierarchical heat map (Figure 2B,C). A multi-step approach was applied to analyze these differentially expressed mRNAs and miRNAs (Figure 3).

### 2.3. Prediction and Selection of miRNA Target Genes

The predicted target genes of deregulated miRNAs were listed using miRWalk 2.0 (http://zmf.umm.uni-heidelberg.de/apps/zmf/mirwalk2/) (2078 predicted target genes of 3 up-regulated miRNAs and 1671 predicted target genes of 3 down-regulated miRNAs) (Tables S3 and S4). Then only genes that were inversely correlated in expression with the differentially expressed miRNAs were selected. This resulted in the selection of 19 target genes of up-regulated miRNAs and only 1 target gene of down-regulated miRNAs (Table 1).

### 2.4. Gene Ontology (GO), Kyoto Encyclopedia of Genes and Genomes (KEGG) Pathway, and Protein-Protein Interaction (PPI) Network Analysis in Differentially Expressed mRNAs

**Figure 4:**
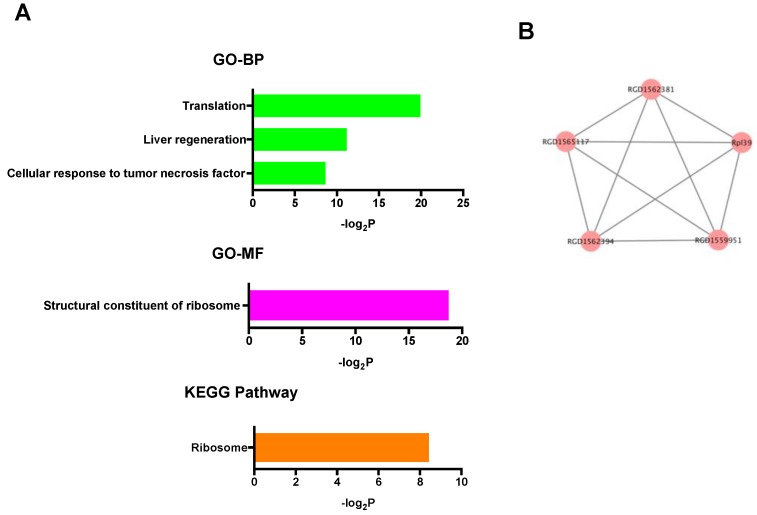
(**A**) GO and KEGG pathway analyses of the up-regulated mRNAs. (**B**) PPI network analysis among the up-regulated mRNAs. Red color node indicates up-regulation. The edges represent the relationships between genes. BP: biological process, GO: gene ontology, KEGG: Kyoto Encyclopedia of Genes and Genomes, MF: molecular function, PPI: protein-protein interaction.

**Figure 5:**
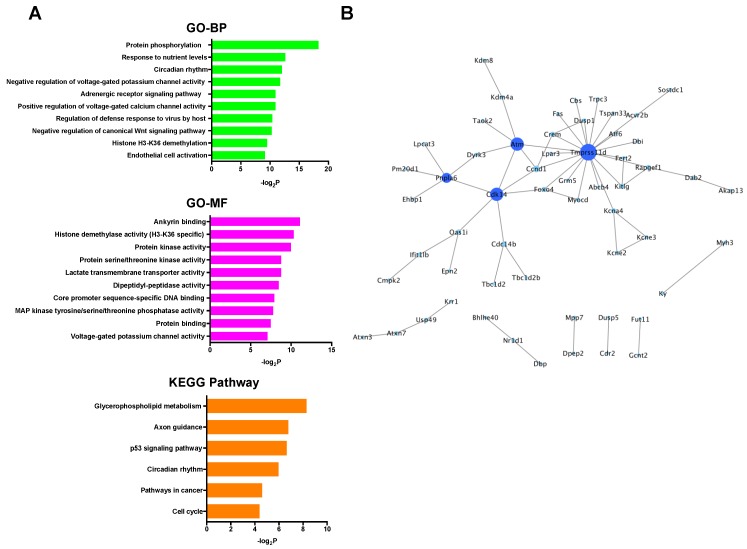
(**A**) GO and KEGG pathway analyses of the down-regulated mRNAs. (**B**) PPI network analysis among the down-regulated mRNAs. Blue color node indicates down-regulation. The size and color depth of nodes represent the number of interactions and the edges represent the relationships between genes. BP: biological process, GO: gene ontology, KEGG: Kyoto Encyclopedia of Genes and Genomes, MF: molecular function, PPI: protein-protein interaction.

In the up-regulated mRNAs, GO terms associated with ribosomal function were mainly enriched, and only the ribosome pathway was significantly enriched in KEGG pathway analysis (Figure 4A). PPI network analysis showed that all selected proteins were ribosomal proteins (Figure 4B). On the other hand, in the down-regulated mRNAs, GO analysis revealed many kinds of significantly enriched GO terms associated with protein phosphorylation and cardioprotection including protein phosphorylation, protein kinase activity, protein serine/threonine kinase activity, and MAP kinase tyrosine/serine/threonine phosphatase activity. Among them, Dusp1 was significantly associated with MAP kinase tyrosine/serine/threonine phosphatase activity. In KEGG pathway analysis, specifically, the p53 signaling pathway, which was previously reported to be associated with cardioprotection against IR injury [36], was significantly enriched (Figure 5A). PPI network analysis revealed 4 hub genes (deep blue nodes) including *Atm*, which has been considered to be an important regulator of the p53 signaling pathway [37] (Figure 5B, Table 2).

### 2.5. GO, KEGG Pathway, PPI Network, and miRNA/mRNA Integrated Network Analysis in Differentially Expressed miRNAs and Selected Target Genes

**Figure 6:**
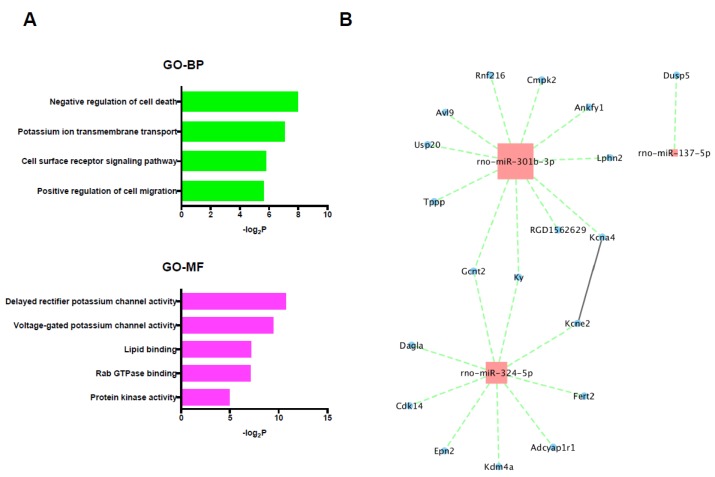
(**A**) GO and KEGG pathway analyses of the selected target mRNAs of the up-regulated miRNAs. (**B**) PPI network and miRNA/mRNA integrated analysis among the selected target genes of up-regulated miRNAs. Red color node indicates up-regulation and blue color node indicates down-regulation. The size of nodes represents the number of interactions. The solid line represents the interaction between genes and the dotted line represents the interaction between miRNAs and the target genes. BP: biological process, GO: gene ontology, MF: molecular function, PPI: protein-protein interaction.

In the 19 selected target genes of the 3 up-regulated miRNAs, specifically, GO terms including negative regulation of cell death and protein kinase activity were significantly enriched; however, no significantly enriched KEGG pathway was detected (Figure 6A). The miRNA/mRNA integrated network analysis showed only 2 mRNAs that were regulated by multiple miRNAs and no hub genes, and only one significant connection between proteins was found in the PPI network analysis using selected target genes of up-regulated miRNAs (Figure 6B).

Regarding the target genes of the 3 down-regulated miRNAs, only 1 mRNA was identified as a selected target gene. Because of the small number of selected target genes, the mRNAs were considered to be unlikely to play an important role in cardioprotection and further bioinformatics analysis was not performed.

### 2.6. Validation of the Results of Microarray Analysis by Real-Time Quantitative Reverse Transcription-Polymerase Chain Reaction (qRT-PCR)

**Figure 7:**
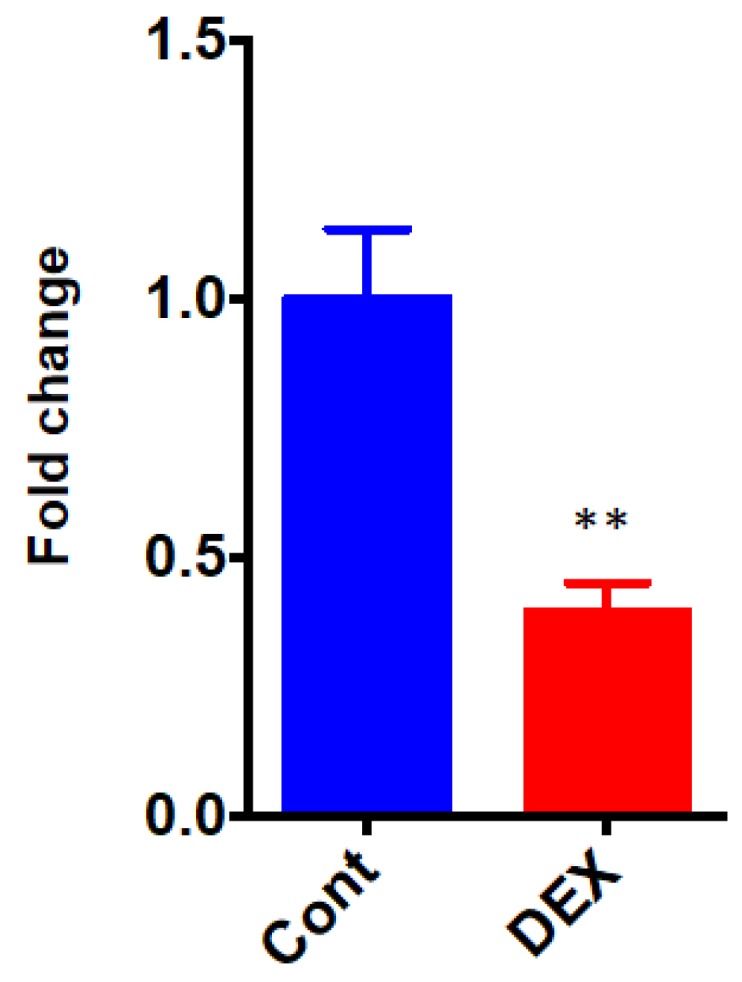
qRT-PCR of Dusp1. Cont: control group, DEX: dexmedetomidine group. *N* = 5 in each group. ** *p* < 0.01.

The differential the of *Dusp1*, a protein phosphatase that dephosphorylates MAPK, was validated by qRT-PCR. The expression level of *Dusp1* was significantly down-regulated after administration of DEX (0.40 ± 0.06 in the DEX group vs. 1.00 ± 0.13 in the Control group, *p* = 0.0031) (Figure 7).

## 3. Discussion

In the present study, differentially expressed mRNAs and miRNAs after DEX administration in rat hearts were successfully identified by conducting microarray analysis. Furthermore, bioinformatics analysis indicated the potential key genes and pathways that might play important roles in the cardioprotective effect of DEX.

Most of the 165 differentially expressed mRNAs were down-regulated (151 genes). Through GO analysis and pathway analysis using these down-regulated mRNAs, multiple GO terms that are associated with protein phosphorylation, MAPK activity, and cardioprotection and KEGG pathways including the p53 signaling pathway, that has been reported to be strongly associated with cardioprotection against IR injury [36], were significantly enriched. Since the ERK1/2 signaling pathway, which is one of the most traditional MAPK signaling pathways, is considered to be one of the mechanisms of cardioprotection induced by DEX [14], the results of the present study reinforce these previous findings. Although it has been shown that direct inhibition of p53 mediates the cardioprotective effect against IR injury [36], no previous study has shown an association between p53 inhibition and DEX induced cardioprotection. Our results indicate that p53 inhibition may be a novel mechanism of the cardioprotective effect of DEX.

We identified 4 hub genes through PPI network analysis using down-regulated mRNAs. Among them, *Atm* might play an important role in DEX-induced cardioprotection. *Atm* is a serine/threonine kinase and activates p53 via phosphorylation of serine residue 15 of p53 [38]. Since it has been shown that administration of a p53 inhibitor prior to ischemia decreased IS in rat hearts [36], down-regulation of *Atm* by DEX might also contribute to cardioprotection. Additionally, we consider that *Dusp1*, a protein phosphatase that dephosphorylates MAPK followed by inhibition of MAPK activities [39], might be a key gene for DEX-induced cardioprotection, although *Dusp1* was not regarded as a hub gene in our PPI network analysis. *Dusp1* has a significant GO term of MAP kinase tyrosine/serine/threonine phosphatase activity, and inhibition of *Dusp1* could be associated with cardioprotection via activation of the MAPK pathway, which is considered to be one of the main mechanisms of DEX-induced cardioprotection [14]. The down-regulation of *Dusp1* in the results of our microarray analysis was validated by the qRT-PCR experiment in the present study. *Tmprss11d*, a serine protease, was the largest hub gene in our PPI network using down-regulated mRNAs. However, there has been no study in which the association between *Tmprss11d* and heart diseases was examined. Further studies are needed to clarify the function of *Tmprss11d* in heart diseases.

By miRNA/mRNA integrated analysis, 19 mRNAs were identified as selected target genes of the 3 up-regulated miRNAs. In GO analysis using these selected target genes, although GO terms of negative regulation of cell death and protein kinase activity were significantly enriched, no significant KEGG pathway was detected. Considering these results and the very small number of selected target genes, the up-regulated miRNAs seem to be unlikely to play important roles in DEX-induced cardioprotection.

Regarding the up-regulated mRNAs, expression levels of 14 mRNAs were up-regulated after DEX administration. GO terms related to ribosomal function were mainly enriched in the GO analysis, and only the ribosome pathway was significantly enriched in the pathway analysis. Additionally, although no hub genes were identified, all of the genes selected in the PPI network analysis were ribosomal proteins. These findings suggest that up-regulated mRNAs after DEX administration might contribute to the regulation of ribosomal function. Previous studies showed that modification of ribosomal proteins was related to cardiac IR injury and cardioprotective signaling [40,41] and that forced overexpression of ribosomal protein S6 enhanced activation of Akt signaling via phosphorylation of serine residue 473 [41]. Alteration of the expression of ribosomal proteins by DEX might be a target of further investigation as a potential mechanism of DEX-induced cardioprotection.

There are some limitations in the present study. First, total RNA was isolated from the heart just after DEX administration before ischemia. The experimental protocol of this study was selected on the basis of results of previous studies showing that cardioprotective signaling was enhanced after DEX administration before the ischemia and reperfusion periods [10,14]. However, different findings might be obtained if samples are harvested at different time points including the early reperfusion phase and end of reperfusion. Second, although we proposed some key genes and pathways that may have critical roles in cardioprotection induced by DEX, whether these genes and pathways directly trigger the cardioprotection induced by DEX was not examined in the present study. Further studies are needed to clarify the causal relationships of these genes and pathways with cardioprotection induced by DEX. Finally, although the differential expression level of *Dusp1* was validated by qRT-PCR in the present study, other significant genes and miRNA-mRNA interactions could be associated with DEX-induced cardioprotection.

In conclusion, we successfully identified differentially expressed mRNAs and miRNAs after DEX administration in rat hearts. Based on the results of bioinformatics analysis, we proposed some possible key genes and pathways that seem to be of significance in DEX-induced cardioprotection, although miRNAs seem to be unlikely to contribute to cardioprotection. Our results might contribute significantly to future investigations aimed at elucidating the mechanism of DEX-induced cardioprotection and establishing a new cardioprotective strategy during the perioperative period.

## 4. Materials and Methods

### 4.1. Animals

All animal experiments were approved by the Institutional Animal Care and Use Committee of Sapporo Medical University (No. 17-114, 14 November 2017) and adhered strictly to the Guidelines for Proper Conduct of Animal Experiments (Science Council of Japan, 1 June 2006). 

Experiments were conducted in male Wistar rats weighing between 250 g to 350 g. The rats were housed in our institutional animal facility in a temperature-controlled room (22–24°C) under a 12-h light/12-h dark cycle with free access to food and water.

### 4.2. Myocardial Ischemia/Reperfusion Injury Model

Rats were anesthetized with 5% sevoflurane and 2.5 mg/kg of butorphanol (i.p.), and anesthesia was maintained with 2% sevoflurane. The trachea was intubated with a 16 G cannula, and the rats were ventilated using a volume-controlled model 683 Rodent Respirator (Harvard Apparatus, Halliston, MA, USA) with tidal volume set to 1 mL/100 g at 60 strokes/min. The body temperature of rats was maintained at 36.5°C–37.5°C with a heat pad. After cannulation of the tail vein with a 24G venous cannula, a left thoracic incision was made in the fourth intercostal space to visualize the left anterior descending coronary artery (LAD). A 6-0 prolene suture was placed around the LAD at 2–3 mm from the tip of the left atrial appendage and the LAD was temporarily ligated by snaring the prolene suture with a PE10 tube for 25 min followed by 60-min reperfusion. Successful ischemia and reperfusion were confirmed by visual inspection of cyanosis over the left ventricular anterior wall and immediate hyperemia after loosening the ligation, respectively. An additional 2.5 mg/kg of butorphanol (i.p.) was administered for postoperative analgesia. Heart rate (HR) and mean arterial blood pressure (MAP) were measured by the tail-cuff method [42] using an MK-1030 blood pressure monitor (Muromachi Kikai, Tokyo, Japan) at 4 time points (baseline, end of DEX preconditioning, end of ischemia, and after reperfusion).

### 4.3. Experimental Protocols

DEX (Maruishi Pharmaceutical, Osaka, Japan) was dissolved in normal saline (NS) and administered intravenously via a 24G venous cannula inserted into the tail vein. The dose of DEX was determined according to a previous study [14] and the manufacturer’s recommendation.

Figure 1A shows the I/R experimental protocol. Rats were randomly assigned to the following experimental groups: (1) a control group (*n* = 7) in which NS was infused at the same rate as that in the DEX group throughout the procedure and (2) a DEX group (*n* = 7) in which DEX was administered at 6 mcg/kg/h (60 mL/kg/h) for 10 min (loading dose) followed by 0.7 mcg/kg/h (7 mL/kg/h) for 20 min (maintenance dose) before ischemia and then NS was infused at 7 mL/kg/h throughout the procedure. In each group, after 30 min of NS or DEX administration and 5 min of washout, 25 min of regional ischemia was applied by LAD ligation followed by 60 min of reperfusion.

Figure 2A shows the experimental protocol for analysis of differentially expressed mRNAs and miRNAs. Rats were randomly assigned to the following experimental groups: (1) a control group (*n* = 5) in which NS was infused at the same rate as that in the DEX group throughout the procedure and (2) a DEX group (*n* = 5) in which DEX was administered at 6 mcg/kg/h (60 mL/kg/h) for 10 min (loading dose) followed by 0.7 mcg/kg/h (7 mL/kg/h) for 20 min (maintenance dose) and then NS was infused at 7 mL/kg/h for 5 min of washout.

### 4.4. Infarct Size (IS) Determination

IS was assessed by the triphenyltetrazolium chloride (TTC) staining technique as previously described [10]. In brief, after the reperfusion period (Figure 1a), the LAD was ligated again and 1.5 mL of 4% Evans blue was injected into the coronary artery via the aortic root to delineate the area at risk (AAR). After that, the heart was rapidly excised and the left ventricle was frozen. The frozen left ventricle was cut into 2-mm-thick slices. The slices were incubated in 1% TTC solution for 15 min at 37 °C and then they were placed in 10% formaldehyde solution for 20 min. LV areas, AAR, and IS were determined by planimetry with ImageJ (National Institutes of Health, Bethesda, MD, USA), and IS was expressed as percentage of AAR.

### 4.5. Total RNA Isolation

After administration of DEX (Figure 2a), total RNA including small RNA was extracted from the left ventricular anterior wall of each rat heart using an miRNeasy Mini Kit (Qiagen, Valencia, CA, USA) according to the protocol of the manufacturer. The concentration and purity of extracted RNA were assessed by using a Nano Drop 1000 Spectrophotometer (Thermo Fisher Scientific, Waltham, MA, USA) and Agilent 2100 Bioanalyzer (Agilent Technology, Santa Clara, CA, USA). A260/A280 > 1.8 and RNA integrity number (RIN) > 7.0 were considered pure and suitable for further analysis.

### 4.6. mRNA and miRNA Microarray Experiments

Microarray analysis was performed with a 3D-Gene Rat Oligo chip 20k (Toray Industries Inc., Tokyo, Japan). For efficient hybridization, this microarray uses a columnar structure to stabilize spot morphology and to enable agitation of microbeads. Total RNA was labeled with Cy5 by using the Amino Allyl MessageAMP II aRNA Amplification Kit (Applied Biosystems, Carlsbad, CA, USA.). The Cy5-labeled aRNA pools were mixed with a hybridization buffer and hybridized for 16 h. The hybridization was performed according to the supplier’s protocols (www.3d-gene.com). Hybridization signals were obtained by using a 3D-Gene Scanner (Toray Industries Inc., Tokyo, Japan) and processed by 3D-Gene Extraction software (Toray Industries Inc., Tokyo, Japan). Detected signals for each gene were normalized by the global normalization method (the median of the detected signal intensity was adjusted to 25).

For miRNA microarray analysis, extracted total RNA was labeled with a 3D-Gene miRNA labeling kit (Toray Industries Inc., Tokyo, Japan). Labeled RNAs were hybridized onto a 3D-Gene Rat miRNA Oligo chip (Toray Industries Inc., Tokyo, Japan). The annotation and oligonucleotide sequences of the probes conformed to miRBase (http://microrna.sanger.ac.uk/sequences/). After stringent washes, fluorescent signals were scanned by the 3D-Gene Scanner (Toray Industries Inc., Tokyo, Japan) and analyzed by using 3D-Gene Extraction software (Toray Industries Inc., Tokyo, Japan). A relative expression level of a given miRNA was calculated by comparing the signal intensities of the valid spots throughout the microarray experiments. The data were globally normalized per array, so that the median of the signal intensity was adjusted to 25.

Differential analysis was carried out using the t-test by Excel (Microsoft, WA, USA). Differentially expressed mRNAs and miRNAs in the control group and DEX group were identified using parameters of *p* < 0.05 and at least 1.5-fold change.

The original microarray data are available at NCBI Gene Expression Omnibus with GEO Series accession number GSE126104 and GSE126105.

### 4.7. miRNA Target Prediction and Selection

First, the targets of differentially expressed miRNAs were computationally predicted by miRWalk 2.0 (http://zmf.umm.uni-heidelberg.de/apps/zmf/mirwalk2/) using three algorithms (miRWalk, MiRanda, Targetscan). Genes predicted by all three algorithms with *p* < 0.05 were identified as predicted target genes [43]. The predicted target genes were compared with the mRNA microarray data, and genes that are inversely correlated in expression with the differentially expressed miRNAs were identified as selected target genes (Figure 3).

### 4.8. Gene Ontology (GO) and Kyoto Encyclopedia of Genes and Genomes (KEGG) Pathway Analysis

GO and KEGG pathway analysis was performed using the Database for Annotation, Visualization, and Integrated Discovery (DAVID) 6.8 online tool [44]. Fisher’s exact *p* value < 0.05 and number of enriched genes >2 were used to identify the significant GO terms and KEGG pathways, and the top 10 GO terms were selected.

### 4.9. Protein-Protein Interaction (PPI) Network

To investigate the functional associations between the proteins coded by the differentially expressed genes, a PPI network was constructed using Search Tool for the Retrieval of Interacting Genes/Proteins (STRING) Version 10.5 (https://string-db.org/) [45]. All of the parameters were included in active interaction sources, and proteins with an interaction score > 0.4 were selected. Genes with a connectivity degree of ≥5 were defined as hub genes. The interactions were visualized using Cytoscape software version 3.7.0 (http://cytoscape.org) [46]. Only proteins that had at least one connection to others were visualized. GO, KEGG pathway, and PPI network analyses were conducted for all of the differentially expressed mRNAs and selected target mRNAs (Figure 3).

### 4.10. Construction of An miRNA/mRNA Integrated Network

Interactions between the differentially expressed miRNAs and the selected target genes were visualized by constructing an miRNA/mRNA integrated network using Cytoscape software version 3.7.0 (http://cytoscape.org) [46].

### 4.11. Real-Time Quantitative Reverse Transcription-Polymerase Chain Reaction (qRT-PCR)

qRT-PCR was performed to validate the results of the microarray experiments [47]. Extracted total RNA was reverse-transcribed to complementary DNA by an miScript II RT Kit (Qiagen, Valencia, CA, USA) using HiFlex buffer according to the manufacturer’s protocol. qRT-PCR was conducted using QuantiTect SYBR Green PCR Kit (Qiagen, Valencia, CA, USA) and the StepOnePlus RT-PCR system (Thermo Fisher Scientific, Waltham, MA, USA) according to the manufacture’s protocol. The IDs of QuantiTect Primer Assays used in the present study were QT00430787 (*Dusp1, NM_053769*) and QT00199633 (*Gapdh, NM_017008*). The expression level of *Gapdh* was used for normalization of mRNA expression levels. The delta-delta cycle threshold method was used to quantify mRNA.

### 4.12. Statistical Analysis

Data are shown as mean ± standard error of the mean. Sudent’s t-test was used to compare two independent groups. Hemodynamic parameters were analyzed using repeated measures 2-way analysis of variance followed by Tukey’s post hoc test. Statistical analyses were performed using GraphPad Prism 6.0 (GraphPad Software, La Jolla, CA, USA). Statistical differences were considered significant with a value of *p* < 0.05.

## Figures and Tables

**Table 1:** Differentially expressed miRNAs and the selected target genes.

**Up-Regulated miRNAs**	**Fold Change**	***p*-Value**	**Selected Target Genes**
rno-miR-137-5p	1.77	0.020	*Dusp5*
rno-miR-301b-3p	1.51	0.039	*Ankfy1, Gcnt2, RGD1562629, Kcna4, Cmpk2, Ky, Avl9, Rnf216, Lphn2, Tppp, Usp20*
rno-miR-324-5p	1.51	0.003	*Adcyap1r1, Fert2, Cdk14, Epn2, Kdm4a, Dagla, Gcnt2, Ky, Kcne2*
**Down-Regulated miRNAs**	**Fold Change**	***p*-Value**	**Selected Target Genes**
rno-miR-34b-3p	0.59	0.007	-
rno-miR-155-3p	0.60	0.041	*Fam219a*
rno-miR-1298	0.67	0.043	-

**Table 2:** Hub genes and the associated significant GO terms and KEGG pathways.

Gene Symbol	Degree	GO-BP	GO-MF	KEGG Pathway
*Tmprss11d*	20	-	-	-
*Atm*	6	Protein phosphorylation	Protein kinase activity Protein serine/threonine kinase activity	p53 signaling pathway Cell cycle
*Cdk14*	6	Protein phosphorylation	Protein kinase activity	-
*Pnpla6*	5	-		Glycerophospholipid metabolism

GO: gene ontology, BP: biological process, MF: molecular function, KEGG: Kyoto Encyclopedia of Genes and Genomes.

## Supplementary Materials

Supplementary materials can be found at https://www.mdpi.com/1422-0067/20/7/1614/s1.
